# Case Report: Secondary bilateral parkinsonism and dystonia treated with dronabinol

**DOI:** 10.12688/f1000research.26476.1

**Published:** 2020-09-21

**Authors:** Natalia Szejko, Florian Burger, Victoria Sidoroff, Gregor K. Wenning

**Affiliations:** 1 Department of Neurology, Medical University of Warsaw, Warsaw, 02091, Poland; 2 Department of Bioethics, Medical University of Warsaw, Warsaw, 02091, Poland; 3 Division of Neurocritical Care, Department of Neurology, Yale University, New Haven, Connecticut, 06519, USA; 4 District Hospital Kufstein, Kufstein, Austria; 5 Department of Neurology, Medical University of Innsbruck, Innsbruck, Austria

**Keywords:** dronabinol, secondary parkinsonism, dystonia, intoxication, ischemic stroke

## Abstract

Drug abuse may damage basal ganglia that are essential for planning and execution of movements. We report a 38-year old patient with ischemic lesions of the basal ganglia presenting with bilateral painful dystonia and parkinsonism caused by polyintoxication. Dronabinol resulted in improvement of pain and gait disturbance, suggesting a novel therapeutic strategy in these challenging patients.

## Introduction

Drug abuse is an important health issue, not only affecting the social surrounding of a patient but also the cerebral integrity. Besides a number of different somatic abnormalities provoked by an intoxication, certain drugs may damage areas of the brain, such as basal ganglia or cortex, which are essential for the movement onset and coordination ^1, 2^.

In this case report, we present a 38-year-old male patient with a long-lasting drug history, including the abuse of cocaine, cannabinoids, benzodiazepines, opiates, methadone and amphetamines. As a result of the multi-intoxication induced bilateral ischaemic lesions to the basal ganglia, the patient presented with secondary bilateral dystonia and parkinsonism. It is not certain whether the cause of ischemia could be related to any specific drug or combinations thereof. Cocaine and its metabolites are known to cause cerebral vasospasm that could lead to ischemic infarctions in the whole brain ^1, 3, 4^ or can provoke haemorrhagic stroke ^2^. Heroin causes ischemia more often in the globus pallidus ^5^. Amphetamines are known as the second most frequent cause of ischemia after cocaine, especially in younger patients due to their vasoconstrictive effect ^1, 6^. Although it is not clear whether cannabinoids and its metabolites can cause cerebrovascular events, there is evidence that cannabis can increase the risk for haemorrhagic stroke ^1, 7^. Opioids may not have a direct toxic impact on the neurons, but ischaemic lesions or necrosis can be triggered by recurrence of drug-induced hypoxia ^1^. 

The aim of this case report is to show the possible use of dronabinol for a multi-intoxicated patient with ischemia of the basal ganglia in order to temper his pain and improve gait.

## Case report

**Figure 1.**
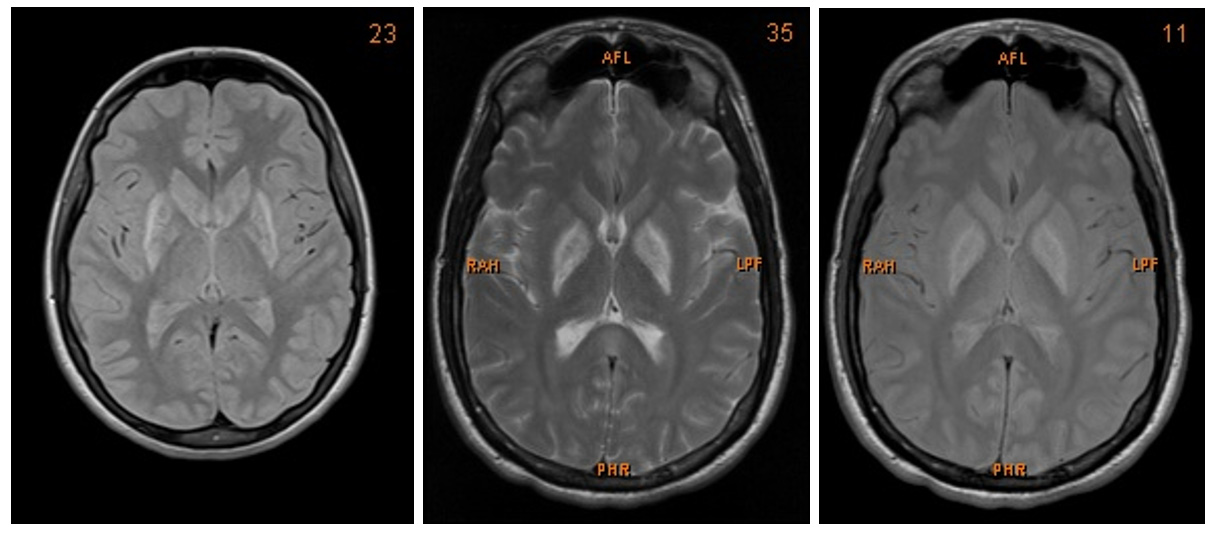
MRI images showing bilateral post-ischaemic lesions of the basal ganglia.

We present a case of a 27-year-old Caucasian man who was admitted to the Intensive Care Unit (ICU) after multi-intoxication. Drug screening on admission identified the following substances: opiates, benzodiazepines, cannabinoids, crack cocaine, methadone and amphetamine. Due to the history of drug abuse he was unemployed. During the first days the patient was in coma, with Glasgow Coma Scale of 5 points. After slow amelioration of his status he presented with severe dysarthria, dysphagia, as well as bilateral parkinsonism and dystonia of his extremities. The neurological examination showed a bilateral positive Babinski sign, global rigidity in all extremities as well as symmetrical hyperreflexia. Furthermore, he suffered from high fever that resolved after treatment with benzodiazepines. Therefore, vegetative symptoms accompanying the patient on submission were interpreted as drug withdrawal syndrome. Both, CT and MRI showed bilateral hypoxemic infarction of the basal ganglia and boundary zone ( Figure 1). Those changes could be the consequence of the mixed intoxication or could be attributed to only one harmful substance, especially crack cocaine or amphetamine. During the hospitalization he developed a tracheobronchitis and respiratory insufficiency due to acute respiratory distress syndrome (ARDS). Moreover, because of severe dysphagia he was nourished via percutaneous endoscopic gastrostomy. Further consequences of his primary condition included post ischemic epilepsy treated with levetiracetam (1000mg). After six months of intensive care, the patient was discharged from hospital. 

During the next five years the patient was treated with levodopa (titrated up to 800mg/day), apomorphine (up to 100 mg/day), selegiline (10mg/day) and baclofen (75mg/day). Further up-titration of levodopa was not possible because of subsequent side effects, such as hallucinations. Due to sleeping problems and agitation he was treated with trazodone (150mg/day) and quetiapine (250mg/day). The substitution dose of buprenorphine 20mg/day was tapered off constantly to keep the patient in a drug naive state. Nonetheless, he showed aggressive behaviour under the influence of alcohol and was admitted to psychiatric ward several times due to delusional disorder and recurrent addictive behaviours. After several months of psychiatric treatment, his addiction and hallucinations resolved. In spite of intense treatment, both parkinsonism as well as dystonia persisted for years and additional symptoms such as generalized pain as well as gait disturbances occurred.

During the examination in our outpatient clinic, 11 years after the hospitalization in the ICU, the patient was still experiencing moderate dysarthria, bilateral dystonia of all extremities, bilateral akinetic-rigid parkinsonism, camptocormia and freezing. Due to those symptoms and increased anxiety as well as painful dystonia as well as unsuccessful treatment with evidence-based agents the patient was treated with dronabinol by his psychiatrist (capsules slowly up titrated to 20mg/day).

After two months of dronabinol treatment, the patient reported subjective improvement of the dystonic pain and a moderate improvement of freezing of gait. We examined the patient at two timepoints: after two months and after two months of dronabinol therapy. Importantly, the medication used at both timepoints remained stable, which excludes confounding contribution of other agents. Results of clinical assessments after two and six months of dronabinol administration are shown in Table 1. The baseline Unified Parkinson’s Disease Rating Scale and Unified Dystonia Rating Scale results cannot be provided as the data were not collected. Although the patient reported the subjective improvement of his symptoms, this was not confirmed in the neurological examination or in the UPDRS and UDRS assessments. Particularly, there was no minimal clinically important differences for UPDRS nor UDRS, although there was an improvement in sleep quality according to ESS. It can therefore be concluded that dronabinol had major analgesic and calming effect and, as a consequence, also improved sleep and general performance. Although he self-reported gait amelioration, it is not clear whether the patient exhibited any motor improvement.

**Table 1.** Results of clinical assessments two and six months after therapy with dronabinol.

Scale	Examination after two months	Examination after six months
UPDRS I	0	0
UPDRS II	18	14
UPDRS III	26	24
UPDRS IV	0	0
H-Y	2.5	2.5
Schwab and England	80%	90%
UDRS	2	2
PDSS-2	1	12
ESS	1	5
BDI	11	10

UPDRS, Unified Parkinson’s Disease Rating Scale; H-Y, Modified Hoehn and Yahr Staging; PDSS-2, Parkinson Disease Sleep Scale-2; UDRS, Unified Dystonia Rating Scale; ESS, Epworth Sleepiness Scale; BDI, Beck Depression Inventory.

## Discussion

While there is some evidence that cannabis-based medicine (CBM) could improve both motor and non-motor symptoms in Parkinson’s disease (PD) ^8, 9^, the effectiveness of CBM in secondary parkinsonism as well as dystonia is unknown. Recently, Peball *et al.* ^10^ reported the potential efficacy of nabilone for PD patients with disturbing non-motor symptoms, which appears to be driven by positive effects on anxious mood and night-time sleep problems. Therefore, CBM might be a useful therapeutic alternative for therapy resistant patients with pain accompanying movement disorders. In the case of our patient, his condition improved only subjectively, but this was not confirmed with objective neurological testing. Moreover, the use of cannabinoids in this case is controversial as the patient had a positive medical history for cannabis misuse. Additionally, long-term effectiveness should also be investigated with accuracy. Potential psychotic effects could be dangerous, therefore the dosage in our patient was slowly increased. Finally, no baseline assessments, prior to dronabinol administration, were available. Results of international randomized, double-blind controlled trials with CBM in PD might offer more scientific rationales for discussion of potential usefulness of CBM in other movement disorders.

## Data availability

All data underlying the results are available as part of the article and no additional source data are required.

## Consent

Written informed consent for publication of their clinical details and clinical images was obtained from the patient.
