# Supplementary material for: Case Report: Secondary bilateral parkinsonism and dystonia treated with dronabinol
Source: F1000Res. 2021 Aug 6;9:1162. Originally published 2020 Sep 21. [Version 3] doi: 10.12688/f1000research.26476.3 (PMC8381340; doi:10.12688/f1000research.26476.3)
Supplement: Peer review history

# Peer review: Reviewer response for version 3

**DOI:** 10.5256/f1000research.59283.r91415

**Published:** 2021-08-20

**Authors:** Giovanni Defazio, Tommaso Ercoli

**Affiliations:** 1 Department of Medical Sciences and Public Health, University of Cagliari, Cagliari, Italy

The authors answered all questions raised by these referees.

Are enough details provided of any physical examination and diagnostic tests, treatment given and outcomes?

Partly

Is the case presented with sufficient detail to be useful for other practitioners?

Yes

Is sufficient discussion included of the importance of the findings and their relevance to future understanding of disease processes, diagnosis or treatment?

Partly

Is the background of the case’s history and progression described in sufficient detail?

Partly

Reviewer Expertise:

Movement disorders

We confirm that we have read this submission and believe that we have an appropriate level of expertise to confirm that it is of an acceptable scientific standard.

# Peer review: Reviewer response for version 2

**DOI:** 10.5256/f1000research.30251.r89189

**Published:** 2021-07-28

**Authors:** Giovanni Defazio, Tommaso Ercoli

**Affiliations:** 1 Department of Medical Sciences and Public Health, University of Cagliari, Cagliari, Italy

The authors reported an interesting and challenging case of secondary bilateral parkinsonism and dystonia. Treatment with dronabinol resulted in improvement of pain and gait disturbance. 

**Major concerns:**

1. Pain improvement was not supported by score change in a specific scale.
2. Likewise, improvement in gait disturbance was not supported by consistent UPDRS/UDRS score change. Subjective improvement of gait was attributed to moderate (subjective?) improvement of freezing of gait, with less falls. However, UPDRS does not assess freezing of gait, and a scale specifically assessing freezing of gait was not administered.
3. The authors should provide a quantitative assessment of the number/time of falls occurred before and after dronabinol.

In conclusion, this report neither support nor discourage the therapeutic use of dronabinol  in secondary movement disorders.

**Minor points:**

 1. Introduction:

 I would suggest changing the introduction starting with a paragraph that briefly describes dystonia and parkinsonism. Some relevant references to be cited:

- Kojovic *et al. *(2013 ^1^)
- Defazio *et al. *(2020 ^2^)
- Korczyn *et al. *(2015 ^3^)
- Belvisi *et al. *(2020 ^4^)

2. Case report:

- Please better describe the neurological examination, in particular please provide more information on dystonia. Was the cranial region involved? Does the patient develop a sensory trick?
- Please correct this typo providing the right timing info: “We examined the patient at two timepoints: after two months and after two months of dronabinol therapy.”

3. Discussion:

- I would suggest adding a short paragraph describing the possible mechanisms of the ischemic event and the relationship between the drug abuse and the MRI findings.

Are enough details provided of any physical examination and diagnostic tests, treatment given and outcomes?

Partly

Is the case presented with sufficient detail to be useful for other practitioners?

Yes

Is sufficient discussion included of the importance of the findings and their relevance to future understanding of disease processes, diagnosis or treatment?

Partly

Is the background of the case’s history and progression described in sufficient detail?

Partly

Reviewer Expertise:

Movement disorders

We confirm that we have read this submission and believe that we have an appropriate level of expertise to confirm that it is of an acceptable scientific standard, however we have significant reservations, as outlined above.

## References

[1] : Secondary and primary dystonia: pathophysiological differences.*Brain*.2013;136(Pt 7) : 10.1093/brain/awt1502038-4910.1093/brain/awt15023771342

[2] : Does acute peripheral trauma contribute to idiopathic adult-onset dystonia?. *Parkinsonism Relat Disord*.71: 10.1016/j.parkreldis.2020.01.00240-4310.1016/j.parkreldis.2020.01.00232007783

[3] : Vascular parkinsonism--characteristics, pathogenesis and treatment.*Nat Rev Neurol*.2015;11(6) : 10.1038/nrneurol.2015.61319-2610.1038/nrneurol.2015.6125917706

[4] : Risk factors of Parkinson disease. *Neurology*.2020;95(18) : 10.1212/WNL.0000000000010813e2500-e250810.1212/WNL.0000000000010813PMC768283332943485

# Response

**Published:** 2021-08-04

**Authors:** Natalia Szejko

**Affiliations:** Department of Clinical Psychiatry, Hannover Medical School, Germany

Thank you so much for reviewing our manuscript. We provide answers to reviewer’s suggestions below:

**Major points:**

- *Pain improvement was not supported by score change in a specific scale.*
- **Response: **We do agree that this would provide more objective results regarding the pain improvement, however, at the moment of examination none pain scales were administered. Unfortunately, we cannot provide these data.
- *Likewise, improvement in gait disturbance was not supported by consistent UPDRS/UDRS score change. Subjective improvement of gait was attributed to moderate (subjective?) improvement of freezing of gait, with less falls. However, UPDRS does not assess freezing of gait, and a scale specifically assessing freezing of gait was not administered.*
- **Response: **We also agree with this suggestion, however, similarly to the previous point, we did not use any scale to assess the freezing of gait.
- *The authors should provide a quantitative assessment of the number/time of falls occurred before and after dronabinol.*
- **Response:** Again, this was not inquired during the patient’s visits, but the patient reported about general improvement in this domain. Moreover, now it is impossible to gain this information due to recall bias.
- *In conclusion, this report neither support nor discourage the therapeutic use of dronabinol  in secondary movement disorders.*
- **Response: **We agree with this statement and reformulated the conclusions appropriately: “All in all, all positive effects were subjective and the final conclusion about the effectiveness of dronabinol cannot be reached”  

**Minor points:**

- *Introduction: I would suggest changing the introduction starting with a paragraph that briefly describes dystonia and parkinsonism. Some relevant references to be cited: Kojovic et al. (2013 ^1^), Defazio et al. (2020 ^2^), Korczyn  et al. (2015 ^3^), Belvisi et al. (2020 ^4^)*
- **Response: **We appreciate this suggestion and have included a new paragraph in the introduction: “However, it is clear that primary and secondary movement disorders have different pathophysiology and, therefore, require different treatments [4-6]”
- *Case report: Please better describe the neurological examination, in particular please provide more information on dystonia. Was the cranial region involved? Does the patient develop a sensory trick?*
- **Response: **We also appreciate this suggestion and have explained it in more detail: “In particular, dystonia was limited to dystonic posturing of the fingers and painful dystonia of the toes.”
- *Please correct this typo providing the right timing info: “We examined the patient at two timepoints: after two months and after two months of dronabinol therapy.”*
- **Resopnse:** We have corrected this typo accordingly and now it states: “We examined the patient at two timepoints: after two months and after six months of dronabinol therapy”.
- *Discussion: I would suggest adding a short paragraph describing the possible mechanisms of the ischemic event and the relationship between the drug abuse and the MRI findings.*
- **Response: **Thank you, we have included paragraph describing possible pathophysiology of these findings: “In case of our patient the suspected mechanism is suspected to be vasospasm provoked by drug abuse that, in turn, provoked ischemia and, finally, abnormal pattern of movements”.

# Peer review: Reviewer response for version 2

**DOI:** 10.5256/f1000research.30251.r73627

**Published:** 2020-10-27

**Authors:** Jose Luis Lopez-Sendon Moreno

**Affiliations:** 1 Neurology Department, Hospital Ramón y Cajal, Madrid, Spain

I believe that the amendments are correct and the article is ready for indexing.

Are enough details provided of any physical examination and diagnostic tests, treatment given and outcomes?

Yes

Is the case presented with sufficient detail to be useful for other practitioners?

Yes

Is sufficient discussion included of the importance of the findings and their relevance to future understanding of disease processes, diagnosis or treatment?

Yes

Is the background of the case’s history and progression described in sufficient detail?

Yes

Reviewer Expertise:

Clinical research. Cannabinoids.

I confirm that I have read this submission and believe that I have an appropriate level of expertise to confirm that it is of an acceptable scientific standard.

# Peer review: Reviewer response for version 1

**DOI:** 10.5256/f1000research.29232.r71804

**Published:** 2020-10-05

**Authors:** Jose Luis Lopez-Sendon Moreno

**Affiliations:** 1 Neurology Department, Hospital Ramón y Cajal, Madrid, Spain

The authors describe an interesting case about a patient with secondary dystonia and parkinsonism and an aparent improvement with dronabinol. 

 The case is relevant since it provides insight into the treatment of a disabling condition. Also, the use of cannabinoids in the setting of movement disorders is a promising field. 

 The case is well described but I have several comments to make: 

- Do the authors have information on other similar cases treated with dronabinol in which no improvement (or perhaps secondary effects) were noted? If so, please comment in order to avoid "publication bias".
- Is the mechanism of action of the parkinsonism isquemic? perhaps the toxicity could be due to other mechanisms (toxic, inflamatory...) Could other "over the counter" substances contribute to the toxicity?
- The authors comment on the lack of structured data on the neurological exploration before treatment. Gait improvement is based on the subjective experience alone. However, could this improvement be quantitatively reflected (Eg. was able to walk unaided, use of support, falls....). If not, then the abstract and conclusions could be overstated. Please correct. 
- The improvement was noted after two months. Please comment why the delay, since the effect should have happened earlier. 
- Some information on the text should be clarified (was the patient six months in intensive care or was that the total time of hospitalization?). 
  As well is repeated twice in the same sentence. Please correct. 
  The examination was done after two months  twice (in the text, not in the table), please correct. 
- There is some evidence (RCT and good reviews) in the use of CBM in mov disorders. Please consider adding further references. 

I believe that the case report should be of interest to the readers and should be indexed with some changes.

Are enough details provided of any physical examination and diagnostic tests, treatment given and outcomes?

Yes

Is the case presented with sufficient detail to be useful for other practitioners?

Yes

Is sufficient discussion included of the importance of the findings and their relevance to future understanding of disease processes, diagnosis or treatment?

Yes

Is the background of the case’s history and progression described in sufficient detail?

Yes

Reviewer Expertise:

Clinical research. Cannabinoids.

I confirm that I have read this submission and believe that I have an appropriate level of expertise to confirm that it is of an acceptable scientific standard, however I have significant reservations, as outlined above.

# Response

**Published:** 2020-10-09

**Authors:** Natalia Szejko

**Affiliations:** Department of Clinical Psychiatry, Hannover Medical School, Germany

Thank you so much for your valuable comments, we would like to address them below. We have also submitted the new version of the manuscript in the effort to address your comments. 

 As for other cases of the movement disorders treated with cannabis based medicine, there is already extensive evidence in this area, reporting also about the side effects. We discuss it in the paper, but have included more references and more extensive description, as suggested.

 When it comes to the exact mechanism of ischemia in this case, it is not entirely clear. However, we suspect it was both toxic and secondary due to vasoconstriction. 

 We have updated the information about the gait improvement, as the patient experienced less falls after treatment with dronabinol. 

 We would like to comment on the time of assessments. The first follow-up was performed after two months and we do not consider it too long, taking into consideration previous reports, this is an adequate time of evaluation. 

 We have checked the manuscript for the timing and updated it accordingly. 

 Finally, we have included more references reporting about the use of CBM in treatment of movement disorders.
